# Is a High HDL-Cholesterol Level Always Beneficial?

**DOI:** 10.3390/biomedicines9091083

**Published:** 2021-08-25

**Authors:** Beata Franczyk, Jacek Rysz, Janusz Ławiński, Magdalena Rysz-Górzyńska, Anna Gluba-Brzózka

**Affiliations:** 1 Department of Nephrology, Hypertension and Family Medicine, Medical University of Lodz, 90-549 Lodz, Poland; beata.franczyk-skora@umed.lodz.pl (B.F.); jacek.rysz@umed.lodz.pl (J.R.); 2 Department of Urology, Institute of Medical Sciences, Medical College of Rzeszow University, 35-549 Rzeszow, Poland; janlaw@op.pl; 3 Department of Ophthalmology and Visual Rehabilitation, Medical University of Lodz, 90-549 Lodz, Poland; mrs-84@wp.pl

**Keywords:** high-density lipoprotein, high levels, dysfunctional HDL, cardiovascular risk

## Abstract

The specific interest concerning HDL cholesterol (HDL-C) is related to its ability to uptake and return surplus cholesterol from peripheral tissues back to the liver and, therefore, to its role in the prevention of cardiovascular diseases, such as atherosclerosis and myocardial infarction, but also transient ischemic attack and stroke. Previous epidemiological studies have indicated that HDL-C concentration is inversely associated with the risk of cardiovascular disease and that it can be used for risk prediction. Some genetic disorders are characterized by markedly elevated levels of HDL-C; however, they do not translate into diminished cardiovascular risk. The search of the potential causative relationship between HDL-C and adverse events has shifted the attention of researchers towards the composition and function of the HDL molecule/subfractions. HDL possesses various cardioprotective properties. However, currently, it appears that higher HDL-C is not necessarily protective against cardiovascular disease, but it can even be harmful in extremely high quantities.

## 1. Introduction

The particular interest concerning high-density lipoprotein (HDL) cholesterol (HDL-C) is associated with its ability to uptake and return surplus cholesterol from peripheral tissues back to the liver and, thus, to its role in the prevention of atherosclerosis, myocardial infarction, transient ischemic attack and stroke [1]. Previous evidence from epidemiological studies has indicated that levels of HDL-C are inversely associated with the risk of cardiovascular disease and that they can be used for risk prediction [2]. The first such findings were demonstrated in the Framingham Heart Study [3]. Therefore, it was concluded that HDL-C is a good carrier of cholesterol that may protect against coronary heart disease. However, interventions aiming at raising HDL-C levels have been shown not to confer better protection against cardiovascular diseases. Moreover, the results of some large studies imply that in some pathological conditions, HDL-C levels do not always correlate with decreased risk of atherosclerosis [1,4]. It has been found that primary familial hyperalphalipoproteinemia, cholesterol ester transfer protein deficiency and endothelial lipase deficiency may be associated with extremely high levels of HDL, but also with, paradoxically, enhanced cardiovascular risk [2].

This review focuses on the reasons and consequences of high and extremely high HDL-C levels in the context of cardiovascular diseases. We searched PubMed using the terms “high and extremely high levels of HDL-C”, “cardiovascular risk”, “U-shaped relationship” and “genetic mutations affecting HDL-C levels” from 2015 to 2021. Relevant articles were selected and their references were also searched for additional relevant articles with no limit on the original date of the article.

## 2. Origin and Properties of HDL Cholesterol

### 2.1. HDL Composition

HDL-C has the highest density of all lipoproteins, as well as the highest proportion of proteins to lipids [1,5]. It is composed of cholesterol, triglycerides, phospholipids and various apolipoproteins, especially Apo-AI (which is the primary structural apolipoprotein of HDL-C and triggers lecithin–cholesterol acyltransferase, LCAT), Apo-AII (acting as an activator of hepatic lipase), Apo-AIV, Apo-AV (activating lipoprotein lipase responsible for triglyceride lipolysis), Apo-CI (activating LCAT), Apo-CII (stimulating LPL), Apo-CIII (responsible for the inhibition of LPL) and Apo-E (serving as a ligand for the LDL receptor) [1]. HDL-proteome was found to comprise 67 proteins involved in cholesterol homeostasis (~50%), including lipid binding (~20%), antioxidant (~6%), acute-phase response (~10–20%), immune response (~1.5%) and endopeptidase/protease inhibition [6]. In pathological states, including acute coronary syndromes, the levels of apoA-IV and haemoglobin beta were demonstrated to be diminished, while levels of serum amyloid A (SAA) and complement C3 (C3) were markedly increased. A higher abundance of SAA, C3 and other inflammatory proteins in HDL-C from patients with ACS may mirror the shift of HDL-C into an inflammatory profile positively affecting the development of the atherosclerotic plaque [6].

### 2.2. HDL Subfractions

HDL particles differ in composition; therefore, there are many various classifications into subfractions obtained using different isolation/separation techniques. Based on the size, HDL particles can be divided into small, medium and large (S, M and L)-HDL subclasses with different chemical and biological properties [7,8]. According to another classification, HDL particles comprise two major subclasses, large buoyant (relatively lipid-rich) HDL2 particles and smaller, denser (relatively protein-rich) HDL3 particles [9,10]. However, using non-denaturing polyacrylamide gradient gel electrophoresis (GGE), HDL2 and HDL3 can be further fractionated in distinct subclasses (HDL3c, HDL3b, HDL3a, HDL2a and HDL2b) with dissimilar electrophoretic mobilities due to different particle size [11]. In turn, the separation based on surface charge and shape resulted in the identification of α-migrating particles (representing the majority of circulating HDL) and preβ-migrating particles (consisting of nascent discoidal and poorly lipidated HDL) [10]. Staining with either Coomassie blue or anti-apolipoprotein A-I (apoA-I) antibodies allows the detection of up to 12 distinct apoA-I-containing HDL subclasses, preβ1 and preβ2, α1, α2, α3, α4 and preα1, preα2 and preα3, according to their mobility and size [10,12,13]. Another recent non-denaturing, linear polyacrylamide gel electrophoresis method enables the separation of 10 HDL subfractions, large buoyant HDL lipoproteins (fractions 1–3), intermediate HDL lipoproteins (4–7) and small-dense HDL lipoproteins (8–10).

### 2.3. The Origins of HDL

The formation of HDL-C starts in the liver and intestine. The first step of HDL-C synthesis comprises the synthesis of its main structural apolipoproteins, Apo-AI. The secretion of lipid-poor protein is followed by the interaction of ApoA-I with the cholesterol–phospholipid transporter ABCA1 (ATP Binding Cassette A1) in order to acquire cholesterol and phospholipids, which results in the formation of nascent HDL-C particle (pre-beta HDL) [14]. In subsequent steps, HDL-C travelling in the circulation gains additional free cholesterol and phospholipids from peripheral tissues, chylomicrons and very-low-density lipoprotein (VLDL) and apolipoproteins coming from the hydrolysis of triglyceride-rich lipoproteins. The core of mature HDL-C particles comprises cholesteryl esters (CE), which are formed by LCAT acting on cholesterol at the surface of HDL-C and subsequently incorporated [15]. Cholesteryl esters in HDL-C can be cleared either via direct uptake by the liver or steroidogenic tissues in a process mediated by HDL-C receptor scavenger receptor B1 (SR-BI) or via plasma cholesteryl ester transfer protein (CETP)–mediated transfer to apoB-containing lipoproteins, typically in exchange for triglycerides. The removal of cholesteryl ester via SR-BI uptake is associated with dissociation and recycling of the smaller apoA-I containing HDL-C particle [16]. In turn, the latter process mediated by CETP results in the depletion of cholesteryl ester from the HDL-C particle, as well as its enrichment in triglyceride. The formation of triglyceride-enriched HDL-C makes it more susceptible to lipolytic modification by hepatic lipase and endothelial lipase, which results in the generation of smaller HDL-C particles subject to faster catabolism. ApoA-I is catabolised in the kidneys [17]. It has been found that HDL-C metabolism (which translates into plasma HDL-C concentration) is mutually regulated by various enzymes, apolipoproteins, cell surface receptors and cellular lipid transporters. The differences in HDL-C particles density, size and composition are associated with the complexity of their metabolism. Observed plasma levels of HDL-C mirror the net state of production, modifications and catabolism [2]. Apart from the aforementioned processes, some genetic and environmental factors can also impact HDL-C levels. For example, the presence of obesity, type 2 diabetes and inflammatory state, as well as smoking, have been demonstrated to decrease HDL-C concentrations, while exercise, oestrogen and thyroid hormone tend to raise its levels [18].

### 2.4. HDL Functions

The primary function of HDL-C involves the transport of cholesterol from the peripheral tissues to the liver [1]. Most cells in peripheral tissues accumulate cholesterol, since they cannot catabolize it; therefore, they require a reverse transport mechanism to return cholesterol to the liver. ABCG1 and ABCA1 transporters enable the transfer of cholesterol to HDL. In subsequent steps, LCAT incorporates this free cholesterol into the HDL-C particle and, ultimately, leads to its uptake in the liver through three distinct pathways (the CETP pathway, the LDL receptor pathway and the SR-B1 pathway) [4,19]. The involvement in such biodistribution of lipids enabling the uptake and return of the cholesterol stored in the foam cells of atherosclerotic plaques to the liver and bile (cholesterol reverse transport), underlines HDL-C anti-atherogenic and anti-inflammatory properties [20,21]. Anti-inflammatory effects of HDL-C are related to its actions leading to the down-regulation of inflammation within the atherosclerotic plaque. Moreover, HDL-C exerts an antithrombotic effect, as well as preventing tumour necrosis factor-alpha (TNF-α)-induced apoptosis of endothelial cells [22,23]. Other atheroprotective properties of HDL-C involve antioxidant effects and NO-promoting effects, as well as anti-apoptotic activities [24,25]. A complementary HDL-C functional capacity covers the ability of this particle to counteract lipid oxidation, especially LDL [26]. Since LDL oxidation is believed to be the prime trigger for the development of atherosclerotic plaques and a crucial promoter of proinflammatory responses in the subendothelial space, the aforementioned HDL-C property is responsible for cardioprotective effects [24]. Endothelial protection is another area covered by the HDL-C particle atheroprotective function [27]. Growing evidence suggests that bioactive lipids containing the HDL-C particle, especially HDL-bound sphingosine 1-phosphate (S1P), could be responsible for such beneficial effects. This modified phospholipid was found to be involved in the increase in nitric oxide production in endothelial cells via the activation of nitric oxide synthase [28]. Moreover, S1P was demonstrated to be a potent chemoattractant for endothelial cells and to limit abnormal vascular permeability via the stimulation of the assembly of vascular endothelial (VE)-cadherin–containing adherens junctions among endothelial cells [29,30,31]. Sattler et al. [32] observed that infusion of S1P restored defective vasodilatory activity of HDL-C from patients with coronary heart disease and the functionality of this lipoprotein. Moreover, 1-SD increment in S1P levels in apolipoprotein B-depleted plasma was found to decrease ACS risk by 30% [26]. Apolipoprotein M (apoM), present primarily in the plasma HDL fraction, is the physiological carrier protein of S1P in HDL [31,33]. The results of studies have indicated that this particle is partly responsible for antiatherogenic effects, probably associated with apoM’s ability to enhance cholesterol efflux from macrophage foam cells, to stimulate preβ-HDL formation, as well as antioxidative properties [31,34,35].

Also, the content of ApoA-I in the HDL-C particle reflects the degree of cardiovascular protection. Soria-Florido et al. [26] revealed that an increase by 1 SD in the level of ApoA-I in apolipoprotein B-depleted plasma decreased almost by half the risk of having ACS. ApoA-I is capable of mediating cholesterol efflux, as well as preventing LDL oxidation by contributing to inactivation and subsequent transfer of lipoperoxides [36,37]. HDL-C may be also involved in the modulation of the immune system via its impact on cholesterol availability in lipid rafts in immune cells and subsequent adjustment of toll-like receptors and MHC-II complex, as well as B- and T-cell receptors [38]; moreover, certain molecules shuttled by HDL-C (e.g., sphingosine-1-phosphate, S1P) were found to contribute to immune cells trafficking. These HDL-C properties have been suggested to be partly related to their pro- and anti-inflammatory properties.

## 3. Conditions Associated with Altered Levels of HDL-C

Genetic disorders associated with markedly increased levels of HDL-C are usually termed primary familial or secondary hyperalphalipoproteinemia (HALP) [39]. According to studies, more than 40 genes impact HDL-C levels [40,41]. Due to the fact that, usually, the presence of such conditions is not associated with visible symptoms, affected patients are identified through the routine assessment of a lipid profile. In patients with primary familial HALP, HDL-C levels exceed the 90th percentile for age and gender despite lack of any secondary causes of elevated HDL-C levels, including medications, malignancies, or liver disease. According to studies, primary familial hyperalphalipoproteinemia is an autosomal-dominant condition that provides protection against atherosclerotic disease and increases the chances of longevity [42]. This condition is associated with the presence of either mutation within the ApoA-I gene, resulting in its overproduction, or variants of apolipoprotein C-III (ApoC-III). ApoA-I protein is not only involved in reverse cholesterol transport (RCT), but it also activates lecithin:cholesterol acyl-transferase (LCAT) and exerts anti-inflammatory effects [43,44]. Therefore, it is believed that ApoA-I overproduction, characterized by increased HDL-C and ApoA-I levels, decreases cardiovascular risk [45]. In turn, a small apolipoprotein ApoC-III regulates plasma triglycerides (TG) homeostasis via inhibiting the activity of the lipoprotein lipase (LPL) and hepatic uptake of TRL by remnant receptors [46,47]. However, ApoC-III not only contributes to elevated plasma TG levels but was also found to promote HDL-C dysfunction, stimulate smooth muscle cell proliferation and facilitate the interaction between monocytes and endothelial cells, as well as alter platelet activity, all triggering atherosclerosis [48]. Thus, mutations that result in the disturbed function of ApoC-III or its loss are associated with significantly decreased TG levels, as well as increased HDL-C concentrations, which translate into a considerable decrease in cardiovascular risk [49,50]. Other causes of HALP involve CETP deficiency. Some polymorphisms within the CETP gene are associated with a reduction in cholesteryl ester transfer activity (mutations of the hydrophobic amino acids at positions 454–475), while others (e.g., Taq1B) affect HDL-C levels in homozygous carriers [51,52]. The CETP enzyme is responsible for the exchange of CE for TG between HDL-C and VLDL/LDL, as well as the regulation of lipid composition and lipoproteins particle size [39,53]. Tall et al. [54] observed that the activity of CETP inversely correlated with HDL-C concentration. CETP deficiency, which is an autosomal recessive inherited metabolic disorder, is associated with increased levels of ApoA-I and ApoA-II resulting from reduced turnover, markedly higher HDL-C levels in homozygotes (usually >100 mg/dL) and moderately raised HDL-C levels in heterozygotes, as a result of lack of HDL-C remodelling [39]. According to estimations, homozygous carriers of loss-of-function mutations in CETP have 80–100% higher levels of HDL-C [55,56]. HDL-C particles of patients with a loss-of-function mutation in CETP are rich in CE and apolipoprotein E (ApoE) and have a low content of TG [45,57]. It seems that CETP deficiency should be beneficial in terms of cardiovascular disease; however, the results of studies provide conflicting data. Some of them indicate reduced CAD risk in such patients, while others suggest that decreased capacity for cholesterol efflux observed in this condition reduces antiatherogenic properties of HDL-C [39,53,58]. Moreover, some mutations in the scavenger receptor class B type I (SR-BI) gene associated with a reduced SR-BI protein expression and function may also alter lipid levels, including HDL-C, in humans [59]. Scavenger receptor class B type 1 is a chief receptor for HDL. SR-BI participates in RCT and mediates the selective uptake of CE from HDL-C in the liver and steroidogenic tissues, as well as enabling the secretion of cholesterol into bile [39]. Some polymorphisms within the SR-BI gene have been demonstrated to increase HDL-C without significantly affecting other lipid measures [60]. Results of studies have indicated that diminished activity or loss of SR-BI function are associated with lower HDL-C bile secretion and consequent higher HDL-C levels. SCARB1 (P376L) SNP was found to be associated with considerably higher HDL-C levels, with a large effect size (beta = 0.22 mmol/L) in more than 300,000 individuals [60]. However, the presence of this mutation also translated into a markedly increased risk of coronary heart disease (CHD) (75% greater odds) in carriers of the P376L allele, compared to non-carrier controls, despite high plasma HDL-C levels. This phenomenon could be related to compromised reverse cholesterol transport resulting from decreased hepatic SR-BI function [60,61]. In addition, SNP within the SCARB1 gene, located within a regulatory enhancer region of SR-BI, was found to increase MI risk. The carriers of the risk C allele had five-fold lower RNA expression of the immune checkpoint inhibitor—lymphocyte activation gene-3 (LAG3)—compared with carriers of the reference G allele [62]. According to Golden et al. [62] plasma sLAG3 protein levels were an independent predictor of HDL-C and increased CVD risk. They observed that, in patients with hyperalphalipoproteinemia (HDL-C ≥ 60 mg/dL), low plasma LAG3 protein levels markedly enhanced MI risk (odds ratio 1.45). Plasma LAG3 was demonstrated to inversely correlate with HDL-C levels (*p*  =  0.007) and IL-10 levels (*p*  <  0.0001).

Finally, abnormally high concentrations of HDL-C could stem from loss-of-function mutations within the endothelial lipase (EL) gene, which is a potent negative regulator of plasma HDL-C [63,64]. Endothelial lipase stimulates HDL-C particle binding and uptake, but it also cleaves HDL-phospholipids releasing fatty acids and lysophospholipids [64]. In case of such disturbances, their impact on cardiovascular risk is unknown. This review focuses on conditions related to high HDL-C; however, it is also worth mentioning that disturbances associated with decreased HDL-C levels do not univocally lead to boosted CAD risk. The results of studies of genetic disorders associated with decreased activity of LCAT and diminished HDL-C levels are often conflicting, in relation to the cardiovascular risk [65]. Sirtori et al. [66] found that carriers of the apolipoprotein A-I(Milano) (apoA-I(M)) mutant, who display very low plasma HDL-C and moderate hypertriglyceridemia, did not suffer from higher cardiovascular risk. Moreover, carriers of the apoA-I(M) with severe hypertension had no structural changes in the arteries and heart, compared to control HA subjects, who showed a significant increase in carotid IMT and higher prevalence of atherosclerotic plaques. In contrast, Hovingh et al. [67], who analysed the apoA-I (L178P) gene defect, revealed lower plasma levels of apoA-I (−50%; *p* < 0.0001) and high-density lipoprotein cholesterol (−63%; *p* < 0.0001) in heterozygotes and these alterations were associated with impaired flow-mediated dilation (FMD) (*p* = 0.012) and elevated carotid intima-media thickness (IMT) (*p* < 0.001), as well 24-fold increase in CAD risk (*p* = 0.003). Therefore, the authors concluded that heterozygosity for this apoA-I mutation triggered a detrimental lipoprotein profile which was related to endothelial dysfunction, augmented carotid arterial wall thickening and markedly boosted CAD risk. Higher risk of premature atherosclerotic cardiovascular disease (ASCVD) was also observed in patients with Tangier disease, a rare disorder related to lack of cellular cholesterol efflux and defects in the ATP binding cassette A1 (ABCA1) transporter and characterized by very low plasma high-density lipoproteins (HDL) levels and cholesterol deposition in various tissues [68,69]. However, some other studies provided conflicting data [70]. The results of the aforementioned and recent studies indicating that HDL-C level is not always the key factor in cardiovascular risk led to a formulation of a new HDL-C function hypothesis. Conditions associated with altered levels of HDL-C are presented in Table 1.

## 4. What Matters in HDL-C Impact on Cardiovascular Risk?

### 4.1. The Role of HDL Components

In 1966, Glomset et al. [71] formulated the concept of reverse cholesterol transport and they suggested that the involvement of HDL-C in this process may be responsible for the protection against coronary heart disease. The results of animal studies have confirmed this thesis. The infusion of HDL-C into cholesterol-fed rabbits was found to inhibit atherosclerosis [72]. In addition, the transgenic mice expressing high amounts of human ApoA-I were demonstrated to be protected from the development of fatty streak lesions while fed with atherogenic diets [73]. Providing that high concentrations of HDL-C are always quantitatively and inversely related to cardiovascular disease, interventions increasing HDL-C levels should bring a significant reduction in its risk. However, many of the performed interventions failed to cause expected effects. A phase 3 clinical trial ACCELERATE (NCT01687998), assessing the effectiveness of the CETP inhibitor, demonstrated that, despite being very effective in increasing HDL-C levels (130% rise in HDL-C), evacetrapib was not superior to placebo in reducing cardiovascular outcomes in patients at high risk for vascular risk [74]. In turn, torcetrapib administration increased levels of high-density lipoprotein cholesterol by 72.1% and decreased LDL (by 24.9%), as compared with baseline; however, in patients receiving this potent CETP inhibitor, enhanced risk of cardiovascular events (HR ratio, 1.25; 95% CI 1.09–1.44; *p* = 0.001) and death from any cause (HR, 1.58; 95% CI, 1.14–2.19; *p* = 0.006) were observed [75]. In addition, in patients with the recent acute coronary syndrome, the CETP inhibitor—dalcetrapib, at a dose of 600 mg daily—increased HDL-C levels but it did not translate into reduced risk of recurrent cardiovascular events [76]. In turn, the results of Randomized EValuation of the Effects of Anacetrapib Through Lipid-modification (REVEAL) clinical trial suggest that the use of anacetrapib lowered the incidence of major coronary events, compared with placebo; however, it is not known whether this effect was associated with the rise in HDL-C cholesterol, the decrease in non-HDL-C, or the concomitant use of statins [77]. The reasons for the discrepant results of trials using CETP inhibitors remains unclear. CETP deficiency was shown to be associated with large and cholesteryl ester-rich HDL-C and polydisperse LDL-C [78]. However, it remains debatable whether such HDL-C reduces or enhances the ability to stimulate cholesterol efflux from macrophages [79,80]. The study assessing the ability of HDL-C obtained from CETP-deficient subjects to protect endothelial cells revealed its markedly lower effectiveness of stimulating NO production than control HDL-C and HDL-C subfractions, as a result of diminished eNOS activating capacity, probably due to decreased S1P content [81]. Gomaraschi et al. [81] also suggested that inhibition of CETP may result in the formation of dysfunctional HDL-C characterized by pro-oxidant and proinflammatory properties. Such dysfunctional particles may also inhibit the eNOS/NO pathway. These findings imply that the presence of CETP deficiency may affect HDL-C structure, thus its function, and it may provide an explanation to why the use of CETP inhibition was not associated with beneficial effects in terms of cardiovascular system protection. Other trials evaluated the efficacy of agents stimulating reverse cholesterol transport, e.g., apoA-I containing recombinant HDL-C particles, or of lipid-poor HDL-C particles. The use of recombinant particles altered in such a way to prevent the apoA-I from being rapidly catabolised was found to exert a favourable impact on atherosclerosis risk [82,83]. The approach involving the infusion of lipid-poor (delipidated) autologous HDL-C was suggested to be associated with some plaque regression in a small study [84]. In addition, the intervention with the use of niacin failed to improve the patients’ outcomes. The administration of niacin accompanying statin therapy brought no clinical benefit during a 36-month follow-up period in patients with atherosclerotic cardiovascular disease, despite substantial improvements in HDL-C and triglyceride levels [85]. It appears that the results obtained from meta-analyses of pharmacological interventions, as well as Mendelian randomization studies, have challenged the thesis that a decrease in cardiovascular risk can be reached by increasing HDL-C levels [26,86,87,88].

### 4.2. HDL-C Levels (U or J Curve)

Moreover, some studies have indicated the loss of protective effect of HDL-C in certain groups of patients, e.g., those with CAD or coronary artery bypass graft surgery [89,90]. Limited data obtained from observational cohort studies have proposed the existence of a plateau effect or elevated CVD risk and higher total mortality in individuals with extremely high HDL-C levels [91,92,93]. Angeloni et al. [89] demonstrated that pre-operative HDL-C levels in patients undergoing isolated first-time elective CABG were not associated with reduced but rather increased major adverse cardiovascular events (MACE) occurrence during follow-up (HR, 1.43; *p* = 0.11). Therefore, they underlined the need for interventions improving HDL-C functionality instead of increasing its levels. In contrast, the analysis of pooled data from six community-based cohorts revealed that the association between HDL-C and CHD events was inverse and linear across most HDL-C values in males and females; however, no further reductions in CHD risk were observed in men with HDL-C values higher than 90 mg/dL and in women with HDL-C exceeding 75 mg/dL [91]. Moreover, the unadjusted models demonstrated increased total mortality risks in men with very high HDL-C, which attenuated after adjustment for traditional risk factors. In addition, in the study of Li et al. [94], a very high level of HDL-C (≥80 mg/dl) was considerably associated with a high risk of all-cause mortality in individuals below the age of 65 years and, in this study, this relationship was independent from other cardiovascular factors. The authors suggested that increasing the level of HDL-C might not guarantee a good prognosis and they also underlined the need to consider the impact of age in HDL-C for mortality risk stratification. Another large-scale pooled analysis of 9 Japanese cohorts including 43,407 participants aged 40–89 years divided into 5 groups differing in HDL-C levels revealed that extremely high levels of HDL-C (≥2.33 mmol/L/≥90 mg/dL) increased the risk of atherosclerotic CVD mortality (hazard ratio = 2.37; 95% confidence interval, 1.37–4.09 for total) and risk for coronary heart disease and ischemic stroke [95]. Moreover, the risk related to extremely high HDL-C was more evident among current drinkers. Some recent studies suggested the existence of a U-shaped curve between HDL-C levels and mortality and morbidity rates of cardiovascular disease [95,96,97]. Therefore, it seems that HDL-C may be a double-edged sword for atherosclerosis [23]. The evidence confirming this thesis comes also from a study demonstrating the impairment of endothelial function in patients with low HDL-C levels, but also in those with extremely high HDL-C concentrations. Moreover, Takaeko et al. [23] demonstrated that, in patients not receiving lipid-lowering therapy, extremely high levels of HDL-C were significantly associated with endothelial dysfunction, after the adjustment for traditional cardiovascular risk factors. The results of combined analysis of 68 long-term prospective cohorts involving over 300,000 individual patient records from across the world confirmed the existence of an inverse linear association between HDL-C and adverse cardiovascular events [98]; however, the division of a studied population into groups based on HDL-C quintiles, revealed the considerable attenuation of the inverse relationship between HDL-C and adverse cardiovascular events in patients with the highest quintile. Another sign suggesting that very high HDL-C may be associated with adverse cardiovascular outcomes comes from the analysis of the Multi-Ethnic Study of Atherosclerosis (MESA) comprising 5500 community-dwelling men and women at low inherent risk for cardiovascular disease [99]. Separate analysis of an HDL-C range of >2.07 mmol/L demonstrated enhanced risk of CHD events (HR, 2.59; 95% CI, 1.11–6.02), compared to the reference HDL-C range. In this study, SNP within SCARB1 was significantly associated with increased subclinical atherosclerosis and incident MI risk. Furthermore, the Cardiovascular Health in Ambulatory Care Research Team (CANHEART) study, analysing data from over 600,000 individuals from Ontario (Canada) who did not suffer from cardiovascular disease, showed a statistically significant rise in the risk of all-cause mortality in males with HDL-C levels >2.07 mmol/L and HDL-C levels <0.78 mmol/L, compared to reference HDL-C levels (1.04–1.30 mmol/L), after the adjustment for covariates, such as heavy alcohol use [100]. Men with low HDL-C (≤30 mg/dL) had particularly enhanced CVD mortality (HR, 1.81; 95% CI, 1.45–2.25), cancer mortality (HR, 1.61, CI, 1.32–1.97) and other mortality HR (HR, 2.01, CI, 1.63–2.47) compared with the reference group (HDL-C, 41–50 mg/dL). Subsequent evidence of the link between very high levels of HDL-C and increased adverse events was obtained in the analysis of two general population cohorts—The Copenhagen City Heart Study and the Copenhagen General Population Study in Copenhagen [97]. These two longitudinal, non-overlapping cohorts of patients with relatively low risk for cardiovascular disease were analyzed together to provide population data from more than 50,000 men and 60,000 women, followed up for a median of 6 years. This analysis revealed a marked ‘U-shaped’ association between HDL-C concentrations and all-cause mortality in both men and women. More pronounced risk of all-cause death was seen in case of HDL-C values exceeding 2.51 mmol/L in men (HR, 1.36; 95% CI, 1.09–1.70) and >3.50 mmol/L in women (HR, 1.68; 95% CI, 1.09–2.58), compared to the lowest risk HDL-C categories for each sex. In this analysis, cardiovascular death was also found to form a U-shaped association with HDL-C values in both men and women. The aforementioned studies included mostly patients at relatively low risk for cardiovascular disease; thus, there are not many data concerning higher-risk individuals. Van der Steeg et al. [92] conducted a post-hoc analysis of two prospective studies, the IDEAL (Incremental Decrease in End Points through Aggressive Lipid Lowering) trial, enrolling nearly 9000 patients with a prior coronary event randomized to receive high-intensity versus moderate-intensity statin therapy, and the EPIC (European Prospective Investigation into Cancer and Nutrition)-Norfolk case-control study, including apparently healthy individuals who did or did not develop CAD during follow-up. In the IDEAL study, elevated HDL-C (<1.04 mmol/L and >2.07 mmol/L versus the lowest risk HDL-C range, 1.55–1.80 mmol/L) was found to be an essential major cardiac event risk factor (such as coronary death, non-fatal MI and resuscitation after cardiac arrest) after the adjustment for age, gender, smoking, apoA-I and apoB [92]. A similar relationship was seen in the case of HDL-C particle size in EPIC-Norfolk. Recently, preliminary data of 5965 participants who had either known CAD or were at high risk for cardiovascular disease, including in the cardiovascular biobank, revealed a “U-shaped” association between HDL-C and CV death/non-fatal MI, as well as all-cause mortality. In this study, individuals with HDL-C <30 mg/dL and ≥60 mg/dL had a much higher risk of all-cause mortality and CV death/non-fatal MI (HR, 1.62; 95% CI = 1.16–2.26, *p* = 0.005, and HR, 1.44; 95% CI = 1.01–2.06, *p* = 0.04, respectively) after adjusting for age, race, sex, body mass index, hypertension, smoking, triglycerides, low-density lipoprotein cholesterol, heart failure history, myocardial infarction (MI) history, diabetes, the use of angiotensin-converting enzyme inhibitor or angiotensin II receptor blocker, beta-blockers, statins and aspirin, as well as estimated glomerular filtration rate and obstructive coronary artery disease [101]. A pooled analysis of data from 37 prospective cohort studies (involving 3,524,505 participants and more than 612,027 deaths) reporting risk estimates of HDL-C levels and mortality demonstrated J-shaped dose-response association between HDL-C level and mortality from all causes, cardiovascular disease and cancer [102]; the lowest risk was observed in patients with HDL-C levels in the ranges of 54–58 mg/dL, 68–71 mg/dL and 64–68 mg/dL, respectively. The pooled hazard ratios for all-cause mortality were 1.03 (95% confidence interval (CI), 1.01, 1.05) and 1.10 (95% CI, 1.09, 1.12), respectively, for each 10-mg/dL increase and decrease in HDL-C levels, compared to an HDL-C level of 56 mg/dL. Thus, the authors concluded that, in the general population, the HDL-C level was associated with mortality from all causes, cardiovascular disease and cancer in a J-shaped dose-response manner, which means that both extremely high and low HDL-C levels are related to an increased risk of mortality [102].

Some discrepancies in the results obtained in the aforementioned studies may be related to the fact that the HDL-C level is affected by demographic and lifestyle factors [88,103].

A possible explanation of the U-shaped relationship between HDL-C and CV risk include genetic mutations leading to very high HDL-C, which also confer adverse CV risk [104]. Alternatively, extremely increased HDL-C may directly represent dysfunctional HDL-C in some individuals, which may, in turn, enhance CV risk. In turn, Feng et al. [105] suggested that free cholesterol transfer to HDL-C upon triglyceride-rich lipoprotein lipolysis by lipoprotein lipase might underlie the U-shape relationship between HDL-C and cardiovascular disease.

Currently, it seems that HDL-C function, rather than its concentration, may be casually related to atheroprotection. It has been found that HDL-C is not a uniform group, but a mixture of subfractions differing in composition and properties. The search of the potential causative relationship between HDL-C and adverse events has shifted the attention of researchers towards the function of the HDL-C molecule/HDL-C subfractions. HDL-C possesses various properties which may be associated with its cardioprotective effects [24]. Scientific interest should shift towards the analysis of numerous HDL-C subspecies and their quality and function to better understand the implications of HDL-C for CV risk assessment [106]. Joint analysis of HDL-C levels stratified by tertiles, as well as its two major subclasses (HDL2-C, HDL3-C), measured in two complementary prospective cohorts—the TRIUMPH study, including 2465 acute myocardial infarction patients, and the IHCS study of 2414 patients who underwent coronary angiography—demonstrated an increase of >50% in both mortality and MI risk in the individuals with lower HDL3-C [107]. However, no significant associations were observed for HDL-C and HDL2-C, which may imply, again, that HDL-C levels alone do not adequately mirror the protective potential of HDL-C against atherogenesis.

The functional features of HDL-C are not as easily measurable as its level. Certainly, the measurement of the functional activity of HDL-C offers a superior ability to predict CV risk, compared to quantitative measurements of HDL-C [106]. Currently, two main validated assays of HDL-C function are available, the cholesterol efflux capacity (CEC) and the HDL-C inflammatory index [108]. The first one is based on a dynamic assessment of the rate and extent (the ability) of HDL-C to remove surplus cholesterol from peripheral cells, primarily macrophages, to the liver [19]. Mature HDL-C is capable of promoting cholesterol efflux mediated by ABCG1, SR-BI and probably other mechanisms [109]. However, the proinflammatory myeloperoxidase may promote oxidative modification and nitrosylation of particular residues on plasma and arterial apolipoprotein A-I in order to render HDL-C dysfunctional, which leads to an increase in cardiovascular risk [110]. Such transformation impairs ABCA1 macrophage transport, activates inflammatory pathways and was found to be associated with an enhanced risk of coronary artery disease. The finding of compromised CEC may reflect the presence of dysfunctional HDL. Patients carrying dysfunctional HDL-C may display a higher risk of atherosclerotic cardiovascular disease [111]. HDL-C becomes dysfunctional in some pathogenic states when it looses anti-inflammatory and antioxidative proteins and, probably, gains proinflammatory ones [111]. Favourable, protective properties of HDL-C seem to be highly dependent on the activity of some enzymes, including paraoxonase-1, HDL-bound phospholipase A2 (HDL-LpPLA2) [26]. In addition, HDL-bound S1P (sphingosine-1-phosphate) is believed to be involved in HDL-C protection on endothelial cells in preclinical models [112]. Moreover, Sattler et al. [113] found that it correlated negatively with the overall severity of CAD and enabled the discrimination between 1-vessel-disease and multi-vessel disease and its low levels appeared to be predictive of CAD extent. Functional properties of HDL-C also depend on relative levels of bound ApoA-I (apolipoprotein A-I) and ApoA-IV (apolipoprotein A-IV) [6]. The enrichment of HDL-C in certain pro-inflammatory proteins, including SAA and C3, was suggested to compromise the antioxidant/anti-inflammatory abilities of the lipoprotein [114]. Soria-Florido et al. [26] demonstrated that impaired HDL-C function, mirrored by reduced CEC and diminished levels of S1P and ApoA-I in apolipoprotein B-depleted plasma contributed to the increase in the risk of ACS, irrespective of HDL-C concentrations and traditional risk factors. Various studies confirmed the inverse association between CEC and adverse cardiovascular events and demonstrated that this measure is a superior marker for incident adverse cardiovascular events, compared to HDL-C levels [115].

The HDL-C inflammatory index is the second functional assessment of this particle in which the degree of LDL oxidation via a cell-free assay (CFA) and LDL mediated monocyte chemotactic activity (MCA) are measured [108]. The presence of pro-oxidant and pro-inflammatory HDL-C particles is associated with higher MCA and CFA values (>1.0), compared to control LDL, while the opposite HDL-C properties reduce these measures below the reference point (1.0). The results of a prospective study performed by Ansell et al. [116] suggested that levels of HDL-C did not affect CFA and MCA values. They found a marked increase in CFA and MCA values in patients with incident CHD and HDL-C values within the normal range, compared to controls, but also in a group of individuals with very high HDL-C values (>2.18 mmol/L), which implies the existence of a dysfunctional form of HDL-C in patients with established cardiovascular disease. Table 2 presents results of RCTs with drugs acting on HDL-C levels.

## 5. Conclusions

Taking into consideration all the above-mentioned results of studies, it appears that higher HDL-C is not necessarily protective against cardiovascular disease and it can even be harmful in extremely high quantities [117]. In addition, the results of some clinical trials showed no benefit of raising HDL-C, which challenged the thesis that increasing plasma HDL-C level uniformly translates into diminished cardiovascular risk [118]. Therefore, current European Society of Cardiology/European Atherosclerosis Society (ESC/EAS) dyslipidaemia guidelines highlight that the risk of atherosclerotic cardiovascular disease may increase when HDL-C levels exceed 90 mg/dL (2.3 mmol/L) [119]. However, the exact reasons for such negative effects of very high levels of HDL-C remain unknown. As already mentioned above, it appears that plasma HDL-C concentration may not be a reliable indicator of the vascular protective function of HDL. In some individuals, extreme raises in HDL-C may reflect the presence of altered HDL-C particles (dysfunctional HDL), which may stimulate the development and/or progression of cardiovascular disease, instead of protecting against it. HDL composition is altered in a complex manner in acute and chronic diseases [120]. The occurrence of genetic mutations leading to very high HDL-C and associated with adverse vascular risk by unknown mechanisms is another plausible explanation of this phenomenon [117]. Thus, it appears misleading to assume that HDL-C is equivalent to “good cholesterol”. Future studies should focus on the identification of pathophysiologically relevant lipids or proteins associated with HDL; currently, such assays are not available for routine clinical use [120].

Current guidelines recommend using the total cardiovascular risk assessment tools while making the decision concerning the primary prevention of cardiovascular disease. Such tools, including the Framingham Risk Score and American College of Cardiology/American Heart Association (ACC/AHA), pooled cohort ASCVD risk calculator, still use HDL-C level as the essential risk measure. Thus, it is apparent that existing risk evaluation tools have not yet been adapted to take into account the current evidence indicating that very high HDL-C fails to protect against atherosclerotic cardiovascular disease. However, recent European guidelines recommend not using HDL-C as a risk measure in cases when HDL-C values exceed 90 mg/L (2.3 mmol/L) [119]. The prognosis concerning cardiovascular disease should not be made based on HDL-C levels. In addition, the HDL-C/LDL-C ratio seems to be misleading in cases of high HDL-C and comorbidities, including CHD, diabetes mellitus and chronic kidney disease [120]. Even though raising HDL-C with the use of drugs is not beneficial, the modification of lifestyle factors, such as physical activity and smoking cessation, was demonstrated to bring vascular protective effects. Currently, the decrease in LDL cholesterol, instead of increasing HDL-C should be a principal goal of lipid therapy.

Future studies should focus on the analysis of HDL-C subclasses that differ in composition and functions. Moreover, emphasis should be placed on the development and standardization of HDL fractionating methodologies, as well as the assessment of their functional properties.

## Figures and Tables

**Table 1:** Conditions associated with altered levels of HDL-C.

Condition	Cause	Findings	Ref.
Primary familial hyperalphalipoproteinemia	Presence of mutation within the *ApoA-I gene* (overproduction) or variants of apolipoprotein C-III (ApoC-III)	✓ Provides protection against atherosclerotic disease and increases the chances of longevity. ✓ Mutations disturbing ApoC-III function, or loss-of-function variations are associated with significantly decreased TG levels and increased HDL-C concentrations, thus considerable decreased cardiovascular risk.	[42,49,50]
CETP deficiency	Loss-of-function mutations in the *CETP gene*	✓ Increased levels of ApoA-I and ApoA-II (reduced turnover). ✓ Markedly higher HDL-C levels in homozygotes (80–100%, higher; usually >100 mg/dL) and moderately raised HDL-C levels in heterozygotes (lack of HDL-C remodelling). ✓ HDL-C particles are rich in CE and apolipoprotein E (ApoE) and have a low content of TG. ✓ Conflicting data concerning the impact of CETP deficiency on cardiovascular disease.	[39,45,55,56,57]
Diminished activity or loss of SR-BI function	Mutations in the scavenger receptor class B type I (*SR-BI) gene*	✓ Reduced SR-BI protein expression and function which alter lipid levels, including HDL-C in humans. ✓ Lower HDL-C bile secretion resulting in higher HDL-C levels.	[59]
	SCARB1 (P376L) SNP SNP within the SCARB1 gene, located within a regulatory enhancer region of SR-BI	✓ Considerably higher HDL-C levels, with a large effect size. ✓ Markedly increased risk of CHD (75% greater odds) in carriers of the P376L allele vs. non-carrier controls, despite high plasma HDL-C levels (compromised reverse cholesterol transport). ✓ Increased MI risk (carriers of the risk C allele had 5-fold lower RNA expression of LAG3, compared with carriers of the reference G allele).	[60,61,62]
Dysfunctional endothelial lipase (EL)	Loss-of-function mutations within the *EL gene*	✓ Abnormally high concentrations of HDL-C.	[63,64]
Apolipoprotein A-I(Milano) mutant	apoA-I(M)	✓ Carriers of apoA-I(M) mutation display with very low plasma HDL-C and moderate hypertriglyceridemia but do not suffer from higher cardiovascular risk. ✓ Carriers of apoA-I(M) with severe hypertension have no structural changes in the arteries and heart, compared to control HA subjects, who showed a significant increase in carotid IMT and higher prevalence of atherosclerotic plaques.	[66]
Defect in apolipoprotein A-I	*apoA-I (L178P) gene*	✓ Carriers have lower plasma levels of apoA-I (−50%; *p* < 0.0001) and HDL-C (−63%; *p* < 0.0001) in heterozygotes. ✓ Mutation associated with impaired FMD (*p* = 0.012), elevated cIMT (*p* < 0.001) and 24-fold increase in CAD risk (*p* = 0.003).	[67]

cIMT, carotid intima-media thickness; EL, endothelial lipase; CHD, coronary heart disease; FMD, flow-mediated dilation; LAG3, lymphocyte activation gene-3.

**Table 2:** Results of RCTs with drugs acting on HDL-C levels.

Study	Intervention	Findings	Ref.
A phase 3 clinical trial ACCELERATE (NCT01687998)	CETP inhibitor (evacetrapib)	✓ Very effective in increasing HDL-C levels (130% rise in HDL-C). ✓ Not superior to placebo in reducing cardiovascular outcomes in patients at high risk for vascular risk.	[74]
A randomized, double-blind study involving 15,067 patients at high cardiovascular risk	Torcetrapib plus atorvastatin, or atorvastatin alone	✓ Increase of 72.1% in HDL-C and decrease of 24.9% in LDL-C, as compared with baseline (*p* < 0.001 for both). ✓ Increased risk of CV events (HR, 1.25; 95% CI, 1.09–1.44; *p* = 0.001) and death from any cause (HR, 1.58; 95% CI, 1.14–2.19; *p* = 0.006).	[75]
A randomized study involving 15,871 patients with a recent acute coronary syndrome	CETP inhibitor (dalcetrapib) at a dose of 600 mg daily, or placebo	✓ Increase in HDL-C levels from baseline by 31–40% in the dalcetrapib group; minimal effect on LDL cholesterol levels. ✓ No change in the risk of the primary end point (cumulative event rate, 8.0% and 8.3%, respectively; HR, 1.04; 95% Cl, 0.93–1.16; *p* = 0.52). ✓ No significant effect on any component of the primary end point or total mortality.	[76]
Randomized EValuation of the Effects of Anacetrapib Through Lipid-modification (REVEAL) double-blind, placebo-controlled clinical trial involving 30,449 adults with atherosclerotic vascular disease	100 mg of anacetrapib once daily, or matching placebo	✓ Increase in mean HDL-C level by 1.12 mmol/L vs. placebo (relative difference of 104%), decrease in mean non-HDL-C level by 0.44 mmol/L (relative difference of −18%). ✓ Decrease in the incidence of major coronary events, compared with placebo (10.8% vs. 11.8%; RR, 0.91; 95% Cl, 0.85–0.97; *p* = 0.004). ✓ Unknown whether this effect was associated with the rise in HDL-C cholesterol, the decrease in non-HDL-C, or the use of statins.	[77]
A randomized placebo-controlled trial conducted at 17 centres in Canada.	4 weekly infusions of placebo (saline), or 40 mg/kg of reconstituted HDL (CSL-111), or 80 mg/kg of CSL-111	✓ The percentage change in atheroma volume was −3.4% with CSL-111 and −1.6% for placebo (*p* = 0.48 between groups, *p* < 0.001 vs. baseline for CSL-111). The nominal change in plaque volume was −5.3 mm^3^ with CSL-111 and −2.3 mm^3^ with placebo (*p* = 0.39 between groups, *p* < 0.001 vs. baseline for CSL-111). ✓ Discontinuation of higher-dosage CSL-111 treatment due to liver function test abnormalities; CSL-111 40 mg/kg resulted in mild, self-limiting transaminase elevation (clinically well-tolerated).	[82,83]
A randomized, placebo-controlled AIM-HIGH clinical trial (NCT00120289) including 3414 patients	Extended-release niacin, 1500–2000 mg/day, or matching placebo; all patients received simvastatin, 40–80 mg per day, plus ezetimibe, 10 mg per day, if needed	✓ At 2 years, a significant increase in median HDL-C level from 0.91 mmol/L to 1.08 mmol/L, decrease in TG level from 1.85 mmol/L to 1.38 mmol/L and reduced LDL-C level from 1.91 mmol/L to 1.60 mmol/L. ✓ The primary end point occurred in 16.4% of patients in the niacin group and 16.2% in the placebo group (HR, 1.02; 95% Cl, 0.87–1.21; *p* = 0.79, by the log-rank test). ✓ No clinical benefit from niacin + statin therapy during a 36-month follow-up period, despite significant improvements in HDL cholesterol and triglyceride levels.	[85]

CETP, cholesteryl ester transfer protein; CI, confidence interval; CV, cardiovascular; HR, hazard ratio; LDL-C, low-density lipoprotein cholesterol; RR, rate ratio; TG, triglyceride.
